# Deep Brain Stimulation and Treatment Outcomes of Young- and Late-Onset (≤55 Years) Parkinson's Disease: A Population-Based Study

**DOI:** 10.3389/fneur.2021.784398

**Published:** 2021-12-10

**Authors:** Emanuele Camerucci, Cole D. Stang, Pierpaolo Turcano, Philip W. Tipton, James H. Bower, Anhar Hassan, Bryan T. Klassen, Rodolfo Savica

**Affiliations:** ^1^Department of Neurology, Mayo Clinic, Rochester, MN, United States; ^2^Department of Neurology, Mayo Clinic, Jacksonville, FL, United States

**Keywords:** Parkinson's disease, young-onset Parkinson's Disease, DBS, Rochester Epidemiology Project (REP), Levodopa

## Abstract

**Background:** No studies have reported the rate of motor complications (MC) and response to medical and surgical treatment in a population-based cohort of young-onset Parkinson's Disease (YOPD) patients and a cohort of sex-matched late-onset Parkinson's Disease (LOPD).

**Objective:** To assess the outcomes of dopaminergic treatment in YOPD and LOPD, explore treatment-induced MC, medical adjustment, and rate of deep brain stimulation (DBS).

**Methods:** We used the expanded Rochester Epidemiology Project (eREP) to investigate a population-based cohort of YOPD between 2010 and 2015 in 7 counties in Minnesota. Cases with onset ≤55 years of age were included as YOPD. An additional sex-matched cohort of LOPD (onset at ≥56 years of age) was included for comparison. All medical records were reviewed to confirm the diagnoses.

**Results:** In the seven counties 2010–15, there were 28 YOPD patients, which were matched with a LOPD cohort. Sixteen (57%) YOPD had MC, as compared to 9 (32%) LOPD. In YOPD, 9 had motor fluctuations (MF) and Levodopa-induced dyskinesia (LID) together, whereas 3 had LID only and 4 MF only. In LOPD, 3 had MF and LID, 3 MF only, and 3 LID only. Following medical treatment for MC, 6/16 YOPD (38%) and 3/9 (33%) LOPD had symptoms resolution. In YOPD, 11/16 (69%) were considered for DBS implantation, in LOPD they were 2/9 (22%), but only 7 (6 YOPD and 1 LOPD) underwent the procedure. YOPD had significantly higher rates in both DBS candidacy and DBS surgery (respectively, *p* = 0.03 and *p* = 0.04). Among DBS-YOPD, 5/6 (83%) had positive motor response to the surgery; the LOPD case had a poor response. We report the population-based incidence of both YOPD with motor complications and YOPD undergoing DBS, which were 1.17 and 0.44 cases per 100,000 person-years, respectively.

**Conclusion:** Fifty-seven percent of our YOPD patients and 32% of the LOPD had motor complications. Roughly half of both YOPD and LOPD were treatment resistant. YOPD had higher rates of DBS candidacy and surgery. Six YOPD and 1 LOPD underwent DBS implantation and most of them had a positive motor response after the surgery.

## Introduction

Treatment-induced motor complications (MC) have been extensively studied in Parkinson's Disease (PD) (1–3). Levodopa is the mainstay treatment for motor symptoms in PD. Unfortunately, although this medication effectively controls symptoms and ameliorates quality of life, motor complications can occur after a few years of treatment with Levodopa (4, 5). These symptoms can be defined as motor fluctuations (MF, namely wearing-off and on-off phenomena) and dyskinesia (6, 7). Motor wearing-off is defined as the re-occurrence of parkinsonian motor features (rest tremor, rigidity, bradykinesia, impaired postural reflexes) that precedes the next dose of Levodopa, indicating that the effect of the medication is fading (1, 8). Some other complications may be unpredictable changes in Levodopa response, when “on” states (this term applies to a period in which Levodopa successfully manages motor symptoms) may be rapidly followed by “off” states (on-off phenomenon) (9).

Dyskinesia describes choreoathetoid movements partially caused by Levodopa therapy and therefore named Levodopa-induced dyskinesia (LID) (10). Nowadays, LID can be classified in peak-dose dyskinesia and diphasic dyskinesia, based on the temporal onset compared to Levodopa dose intake.

Thus, the role of neurologists consists in tailoring Levodopa doses to avoid dyskinesia during “on” periods and Parkinsonism during “off” periods. In order to address this problem, clinicians use a number of strategies, but ultimately surgical procedures are usually performed when the medical adjustments fail. In particular, deep brain stimulation (DBS) represents the most common, safe and effective solution for MF (7, 11). Some of the preferred target brain areas to treat PD are subthalamic nucleus (STN), internal globus pallidus (GPi), and ventralis intermedius (VIM) nucleus (11). Targeting these areas has shown to improve dyskinesia, UPDRS score, Academic Medical Center Linear Disability Scale (ADLS), and Levodopa usage after the surgery (11–13).

Patients with young-onset Parkinson's Disease (YOPD) have been thought to be affected more substantially and earlier by MC because of the possible longer duration of treatment and different response to medications. Therefore, we took advantage of the expanded Rochester Epidemiology Project (eREP) to establish a population-based cohorts of YOPD in Olmsted County (MN) and in the surrounding six counties between 2010 and 15. We also compared the cases of YOPD with a sex-matched cohort of late-onset PD (LOPD) in Olmsted County between 2010 and 2015 regarding dopaminergic treatment, the adjustments needed, and their clinical outcomes.

## Methods

### Case Ascertainment

We used the eREP medical records-linkage system to identify the cases of PD among individuals who reside in Olmsted County, Minnesota, and the six surrounding counties (Dodge, Freeborn, Mower, Steele, Wabasha, and Waseca) (14, 15). This records-linkage system provides the infrastructure for indexing and linking all medical information of the counties' population. All medical diagnoses, surgical interventions, and other procedures are entered into computerized indexes using codes from the Hospital Adaptation of the International Classification of Diseases—9th Revision (H-ICDA) or the International Classification of Diseases—10th Revision (ICD-9).

We ascertained potential cases of Parkinsonism using a computerized screening phase and a clinical confirmation phase. In phase 1, we searched the indexes for 38 diagnostic codes potentially indicative of Parkinsonism, including: 5 codes for PD, 14 for Parkinsonism, 7 for tremor, 2 for extrapyramidal disorders, 5 for non-specific neurodegenerative diseases, 2 for multiple system atrophy, and 3 for progressive supranuclear palsy. This list of 38 codes was designed to yield maximum sensitivity at the cost of low specificity and high false positive rate.

In phase 2, a physician (E.C.) reviewed the complete records of the patients who had a code of interest during 2010–2015 or in the following 3 years to exclude individuals who were false positives. The data collection was performed during summer of 2020 hence patients' follow up ranged from 5 to 10 years. We extended the search for incident cases for 3 years (2016–2018) to ensure that persons with delayed diagnosis could be correctly counted. We then included in this study only those individuals with symptoms onset at 55 years of age or below.

We also established a sex-matched cohort of LOPD by randomly selecting an equal number of PD patients with onset of disease between 2010 and 2015 in Olmsted County (MN). LOPD cases were defined as having disease onset at 56 years of age or greater.

The physician defined the likeliness of diagnosis, the onset date and the type of Parkinsonism. Onset of PD was defined as the approximate date in which 1 of the 4 cardinal signs of PD was first noted by the patient, by family members, or by a care provider (as documented in the medical record). The validity of this approach is discussed and reported elsewhere (16).

### Diagnostic Criteria

Our diagnostic criteria were consisting of two steps: the definition of Parkinsonism as a syndrome and the definition of types of Parkinsonism within the syndrome. Parkinsonism was defined as the presence of at least two of four cardinal signs: rest tremor, bradykinesia, rigidity, and impaired postural reflexes. PD was diagnosed when all of the three following were present: no other causes; no documentation of unresponsiveness to levodopa at doses of at least 1 gm/day in combination of carbidopa; no prominent or early signs of more extensive nervous system involvement (17, 18). Following that, we defined YOPD as a PD with an onset before the 56th birthday, and LOPD has an onset at 56 years or more.

Motor complications were defined as the oscillatory response to Levodopa treatment with sudden off-periods, as defined in the Unified Parkinson's Disease Rating Scale (19), and involuntary movements (peak-dose, diphasic and off-period) related to treatment and their time of first occurrence (20). The presence of fluctuations and dyskinesia was reported when directly assessed by the neurologist or when referred by patients and written in the clinical records.

## Statistical Analysis

We excluded individuals who denied authorization to use their medical records for research. All the subjects with PD onset at or before 55 years of age between January 1st, 2010 and December 31st, 2015, and with residence in any of the seven counties (at symptoms onset) were included as YOPD cases. We calculated incidence using incident cases as numerator and eREP Census (restricted to those with <56 years of age) as denominator. Since our study was descriptive and involved the entire counties population, no sampling procedures were involved and confidence intervals and statistical tests were not necessary for the interpretation of incidence rates (17, 18, 21).

Continuous variables are summarized with medians and interquartile range (IQR); categorical variables are summarized with frequency counts and percentages. Chi-square test was used to compare rates of motor complications, treatment strategies, and DBS candidacy/surgery between the 2 cohorts.

## Standard Protocol Approvals, Registrations, and Patient Consents

This study was approved by the Mayo Clinic and Olmsted Medical Center Institutional Review Boards. Participating patients (or their legally authorized representatives) provided informed written consent for use their medical information for research.

## Results

We observed 28 YOPD cases in seven counties in Minnesota between 2010 and 15 (19 men and 9 women). Median age at YOPD onset was 51.5 years (Q1: 50; Q3: 53); 52 years (Q1: 50; Q3: 53) in men and 50 years (Q1: 50; Q3: 54) in women. We matched them by randomly selecting an equal number of LOPD cases. Sex distribution was set equal as the one in YOPD (19 men and 9 women). Median age at LOPD onset was 71 years (Q1: 66; Q3: 78); 71 years (Q1: 66; Q3: 75) in men and 75 years (Q1: 66; Q3: 79) in women. Demographic information concerning the 2 cohorts are reported in Table 1.

**Table 1:** Demographic information.

	**YOPD**	**LOPD**
Gender	19/9	19/9
Age at onset overall	52 years (Q1: 50; Q3: 53)	71 years (Q1: 66; Q3: 78)
Men	52 years (Q1: 50; Q3: 53)	71 years (Q1: 66; Q3: 75)
Women	50 years (Q1: 50; Q3: 54)	75 years (Q1: 66; Q3: 79)
PD onset in patients with motor complications	50.5 years (Q1: 46.5; Q3: 52.3	66 years (Q1: 60; Q3: 72)
Men	52 years (Q1: 49; Q3: 53)	68 years (Q1: 62; Q3: 72)
Women	47 years (Q1: 46; Q3: 48.5)	66 years (Q1: 63; Q3: 75)
Median age at DBS implantation	57 years (Q1: 56; Q3: 57)	67 years
Men	57 years (Q1: 55; Q3: 58)	–
Women	57 years (Q1: 56; Q3: 57)	67 years
Time from PD onset to DBS implantation	6.8 years (Q1: 5.5; Q3: 7.5)	7.6 years
Men	5.8 years (Q1: 4.7; Q3: 6.7)	–
Women	7.6 years (Q1: 7.6; Q3: 7.6)	7.6 years

Among the 28 YOPD, we observed 16 cases (57%) of MC (13 men and 3 women). According to the clinical notes, there were 4 patients (14%) with MF only, 3 (11%) with LID only, and 9 (31%) with MF and LID.

Median age at YOPD onset among the 16 patients with treatment-induced motor complications was 50.5 years (Q1: 46.5; Q3: 52.3), in men 52 years (Q1: 49; Q3: 53) and in women 47 years (Q1: 46; Q3: 48.5).

Whereas, in the LOPD cohort, we reported 9 patients with MC (32%, 6 men and 3 women). Of these, 3 had MF only, 3 LID only, and 3 had both MF and LID. Median age at LOPD onset among the 9 patients with treatment-induced MC was 66 years (Q1: 60; Q3: 72), in men 68 years (Q1: 62; Q3: 72) and in women 66 years (Q1: 63; Q3: 75).

YOPD and LOPD did not significantly differ in terms of rates of MC (*p* = 0.06), MF only (*p* = 0.68), LID only (*p* = 1.00), and MF and LID (*p* = 0.051).

Treatment outcomes in our YOPD and LOPD cohorts are shown in Table 2.

**Table 2:** Motor complications and treatment outcome in YOPD and LOPD.

	**YOPD**	**LOPD**	***p*-value**
Gender (M/F)	19/9	19/9	–
Motor complications overall	16/28 (57%)	9/28 (32%)	0.06
MF only	4/28 (14%)	3/28 (11%)	0.68
LID only	3/28 (11%)	3/28 (11%)	1.00
MF and LID	9/28 (31%)	3/28 (11%)	0.051
**Medication changes**			
Current medication dose readjustment	10/16 (63%)	7/9 (78%)	0.43
Additional medications added	5/16 (31%)	–	–
No changes in treatment	1/16 (6%)	2/9 (22%)	0.24
Complications resolved?	6/16 (38%)	3/9 (33%)	0.83
Treatment resistant	9/16 (56%)	4/9 (44%)	0.57
Was considered for DBS	11/16 (69%)	2/9 (22%)	0.03^*^
Underwent DBS and had motor complications	6/16 (38%)	1/9 (11%)	0.16
Underwent DBS	6/28 (21%)	1/28 (4%)	0.04^*^
**DBS outcome**			
Poor	1/6 (17%)	1/1 (100%)	–
Modest	2/6 (33%)	–	–
Robust	3/6 (50%)	–	–

* *Statistically significant*.

In YOPD, 16 patients had MC. Their max Levodopa dose was 1,200 mg (Q1: 825; 1,425). Levodopa dose adjustment (including adding a different formulations of carbidopa/levodopa) was tried in 10/16 (63%) cases; Amantadine was prescribed in 2/16 (13%) cases (respectively, 200 and 300 mg per day); in 1 case (6%) motor symptoms were mild and did not require any medications changes.

Among our 16 cases with motor complications, 6 (38%) responded well to medical treatment. These were 3 cases of LID alone (treated readjusting the current Levodopa dosing), and 2 of MF+LID (treated readjusting Levodopa dosage and adding Amantadine for LID).

Nine patients (56%) did not have resolution of their motor complications with medical treatment.

All the patients with motor complications resistant to medical treatment were considered for DBS surgery, plus two additional YOPD patients considered due to treatment-resistant tremor. Among the 11 patients considered for DBS surgery, 6 (55%) underwent the procedure (4 men and 2 women). Main reason for opting out of the surgery was personal (4 cases) and contraindications (1 case). Response to surgery was deemed poor in 1 case (17%), modest in 2 (33%), and robust in 3 (50%).

Between the DBS-YOPD patients, median age at YOPD onset was 49.5 years (Q1: 47.5; Q3: 50.8). Median age at DBS implantation was 56.6 years (Q1: 55.5; Q3: 57.4). All of them had bilateral STN DBS implantation; one of them had a prior bilateral VIM implantation. Implantation occurred 6.8 years (Q1: 5.5; Q3: 7.4) after YOPD onset.

In LOPD, we observed several differences, as compared to YOPD. Nine of them had motor complications (max Levodopa dose among them was 1,000, Q1: 800; Q3: 1,400), 7 of which were treated by simply readjusting the current medication; the remaining 2 did not have bothering symptoms. Hence, a modification of the treatment was not deemed necessary. Among those 7 who had the Levodopa dose readjusted, 3 (43%) reported resolution of their motor symptoms. In those 4 patients where MC were not properly addressed by medications, 2 of them (50%) were considered for DBS implantation, and 1 underwent the surgery (the other one was deemed ineligible due to comorbidities).

Differences between YOPD and LOPD were significantly different only in terms of DBS consideration rate (YOPD 69% and LOPD 22%, *p* = 0.03). No differences were observed in the way MC were addressed and in rate of resolved complications. Also the rate of patients undergoing DBS was not different among individuals with MC (*p* = 0.16), but it was when considering the whole cohort (*p* = 0.04).

### Incidence of YOPD

We reported that 57% YOPD patients had medication-induced motor complications. In particular, in the 2010–2015 cohort of residents in Olmsted and the surrounding six counties (≤ 55 years), incidence of YOPD with motor complications was 1.17 per 100,000 person-years.

Among all the incident cases of YOPD, DBS was required in 6/28 (21%) of them. In the 2010–2015 cohort of residents in the seven counties (≤ 55 years), incidence of DBS-YOPD was 0.44 per 100,000 person-years and it was required in 6 (38%) of the 16 YOPD patients with motor complications.

### Survival

At the time of data collection, only 1 (4%) YOPD patient had died after 1 year from the onset of YOPD onset due to esophageal adenocarcinoma.

Among LOPD, 5 (18%) had died at time of data collection. They were 4 men and 1 woman; they died at a median age of 85 years of age (Q1: 84; Q3: 85) due to carcinomas (2 cases), cardiorespiratory causes (2 cases), and complications of PD (1 case).

## Discussion

In our study using eREP, we report that 57% of our YOPD patients had treatment-induced motor complications. We also had the opportunity to explore the incidence of our 16 YOPD patients with motor complications in our population-based cohort, which was 1.17 cases per 100,000 person-years. Such high incidence was not unexpected, as PD occurring at an earlier age has greater likelihood to develop motor complications (22–25).

Frequency of MC within YOPD trended higher than LOPD [even though *p*-value was proximate to the significance level (*p* = 0.06), a possible effect of the small sample size] and, in general, was higher than the one reported in the available literature in non-age-restricted PD (26, 27), but lower than the other studies exploring MF and LID in YOPD (22, 23, 25), where it was ranging between 70 and 100% (22, 23, 25). The differences observed with other YOPD studies can be imputed to a different cut-off ages used for YOPD definition. We indeed used ≤ 55 years, whereas other authors adopted: <40 years (25), <50 years (22), and ≤ 45 years (23). Nevertheless, using a younger age of onset would result in a higher prevalence of motor complications, because their prevalence decreases as the onset of disease occurs later in life (22, 24, 25, 28).

In patients developing MF and/or LID, several medical strategies should be taken into account. Particularly, in LID, a reduction of dopaminergic doses should not be considered as a viable option, as it will likely worsen Parkinsonism (29). Hence, Amantadine should be used by clinicians because it showed an efficacy ranging between 60 and 70% (30) with a consistent efficacy over time (31). Interestingly, in our cohort, Amantadine was used rather infrequently (only twice among YOPD and never in LOPD) but showed optimal results in terms of LID improvement. Both patients were subsequently considered for DBS surgery because of additional motor symptoms unrelated to LID.

When treating MF, an increase in the dopaminergic doses should be considered (32) by adding new medications, readjusting Levodopa doses, or adding different Levodopa formulations (32). In our cohort, Levodopa dose readjustment was the chosen MF addressing strategy in virtually all cases, but outcomes were mostly negative (among individuals with MF, only 17% had a positive outcome in YOPD and 40% in LOPD).

Seven of our patients underwent DBS implantation (6 YOPD and 1 LOPD) out of a total of 13 (11 YOPD and 2 LOPD) candidates for the procedure. We report a prevalence of DBS surgery of 21% and a prevalence of DBS candidacy of 39% among YOPD; it was 4 and 7% among LOPD, both statistically significant.

The higher prevalence of DBS among YOPD and the differences with LOPD were not unexpected. DBS implantation has been used in younger patients due to the general idea that they might have a lesser burden of comorbidities, less complications, and a better response to external stressors, such as invasive neurosurgical procedures (33, 34). In the recent era, evidence have been gathered supporting the idea that performing DBS implantation at an older age does not increase the risk of complications (33, 35, 36). Thus, age alone is not a reliable exclusion criterion for determining DBS candidacy. A possible inclusion/exclusion factor should be the onset of motor complications: it has been shown that performing such procedure earlier rather than later has greater benefits for the patients (35, 37, 38). Indeed, in our cohort, most of the DBS-YOPD patients had a positive response to DBS implantation, which was deemed robust in 50% of the cases.

Despite these strong evidence hinting toward the benefits of performing DBS at an earlier stage (regardless of patient's age), a recent study in UK (39) showed that, although the number of DBS procedures increased by 26-fold from 1997 to 2012, age at surgery remained constant at 60 years, 11 years after PD diagnosis. Our data suggest that in our clinical practice we tend to perform the surgery slightly earlier in the disease course, given the discrepant prevalence of DBS in YOPD and LOPD and the fact that our YOPD patients underwent DBS implantation at a median age of 56.6 years, almost 7 years after YOPD onset, similarly to the only LOPD case (who underwent surgery at 67 years of age, 7 years after disease onset). Therefore, it is indeed recommended to perform surgery when it is needed without the need of waiting.

We also report that 57% of our patients were men. Even though we had a relatively low sample size of patients undergoing DBS surgery, these results do not differ from the currently available literature. This gender difference may be due to the fact that PD incidence is higher in men (16). Another possible explanation could be that women tend to have a more benign clinical course; hence, they may require surgery less frequently (40).

Our study has several strengths. First, the usage of the medical records-linkage system of eREP provided us with the possibility to access all medical information of the population of Olmsted County and the six surrounding counties in the timeframe chosen. Second, all medical records were reviewed by a physician (E.C.) to confirm the final diagnosis. Third, the standardized codes allowed us to detect all the incident cases of Parkinsonism in predetermined geographical and temporal settings. Fourth, we extended our search for three years following the incidence period in order to capture all cases with onset in the incidence period but with diagnosis in the subsequent years.

We also acknowledge that, due to the rarity of YOPD and the fact that not all of them had MF and/or LID and required DBS implantation, we had a relatively small sample size, which could limit the inference in a larger population. We also acknowledge that the medical records are not standardized for research purposes, which may impact our detection rate of specific clinical information. Lastly, both our YOPD and LOPD cohorts had disease onset between 2010 and 2015, hence their follow up is relatively short, but already provides information about a subtype of patients with YOPD that require DBS early on in their disease course.

## Conclusion

YOPD patients had higher degree of motor complications, as compared to LOPD (57 vs. 32%); of these, 56% YOPD and 57% LOPD did not respond to medical treatment and were considered for DBS implantation. Six YOPD (67%) and 1 LOPD (25%) among the non-responders underwent DBS implantation and most of them had a positive motor response after the surgery. YOPD had significantly higher DBS candidacy (*p* = 0.03) and DBS surgery (*p* = 0.04) as compared to LOPD.

In Olmsted County (MN) and the six surrounding counties 2010–2015 (≤ 55 years), incidence of YOPD patients with motor complications was 1.17 cases per 100,000 person-years, whereas in DBS-YOPD it was 0.44 cases per 100,000 person-years.

## Data Availability Statement

The raw data supporting the conclusions of this article will be made available by the authors, without undue reservation.

## Ethics Statement

The studies involving human participants were reviewed and approved by Mayo Clinic and Olmsted Medical Center Institutional Review Boards. The patients/participants provided their written informed consent to participate in this study.

## Author Contributions

EC: execution of research project and writing the first draft. CS, PT, PWT, and AH: execution of research project and review and critique of manuscript. JB: organization of research project, statistical review and critique, and review and critique of manuscript. BK: organization and execution of research project and review and critique of manuscript. RS: conception, organization of research project, statistical design, review and execution, and review and critique of manuscript. All authors contributed to the article and approved the submitted version.

## Funding

This study was partially funded by Acadia Pharmaceuticals, Inc., and by CTSA (UL1 TR002377) from the National Center for Advancing Translational Sciences (NCATS), a component of the National Institutes of Health (NIH). This study was also supported by the National Institute on Aging of the National Institutes of Health (grant number R01 AG034676) and by the Mayo Foundation for Medical Education and Research. The funders also had no role in the conception, design, or writing of this study.

## Author Disclaimer

The contents are solely the responsibility of the authors and do not necessarily represent the official view of the funders.

## Conflict of Interest

JB receives support from the Parkinson's Disease Foundation. AH receives research support from NINDS and IntraBio, Ltd. BK receives research funding from NINDS, DARPA, and CalaHealth. RS receives support from the National Institute on Aging, the National Institute of Neurological Disorders and Stroke, the Parkinson's Disease Foundation, and Acadia Pharmaceuticals. The remaining authors declare that the research was conducted in the absence of any commercial or financial relationships that could be construed as a potential conflict of interest.

## Publisher's Note

All claims expressed in this article are solely those of the authors and do not necessarily represent those of their affiliated organizations, or those of the publisher, the editors and the reviewers. Any product that may be evaluated in this article, or claim that may be made by its manufacturer, is not guaranteed or endorsed by the publisher.
